# Silicon Application Increases Drought Tolerance of Kentucky Bluegrass by Improving Plant Water Relations and Morphophysiological Functions

**DOI:** 10.1155/2014/368694

**Published:** 2014-06-29

**Authors:** Shah Saud, Xin Li, Yang Chen, Lu Zhang, Shah Fahad, Saddam Hussain, Arooj Sadiq, Yajun Chen

**Affiliations:** ^1^Horticulture College of Northeast Agricultural University, Harbin, Heilongjiang 150030, China; ^2^National Key Laboratory of Crop Genetic Improvement, MOA Key Laboratory of Crop Ecophysiology and Farming System in the Middle Reaches of the Yangtze River, College of Plant Science and Technology, Huazhong Agricultural University, Wuhan, Hubei 430070, China; ^3^Department of Agriculture Resource and Environmental Sciences, Northeast Agriculture University, Harbin 150030, China

## Abstract

Drought stress encumbers the growth of turfgrass principally by disrupting the plant-water relations and physiological functions. The present study was carried out to appraise the role of silicon (Si) in improving the drought tolerance in Kentucky bluegrass (*Poa pratensis* L.). Drought stress and four levels (0, 200, 400, and 800 mg L^−1^) of Si (Na_2_SiO_3_·9H_2_O) were imposed after 2 months old plants cultured under glasshouse conditions. Drought stress was found to decrease the photosynthesis, transpiration rate, stomatal conductance, leaf water content, relative growth rate, water use efficiency, and turf quality, but to increase in the root/shoot and leaf carbon/nitrogen ratio. Such physiological interferences, disturbances in plant water relations, and visually noticeable growth reductions in Kentucky bluegrass were significantly alleviated by the addition of Si after drought stress. For example, Si application at 400 mg L^−1^ significantly increased the net photosynthesis by 44%, leaf water contents by 33%, leaf green color by 42%, and turf quality by 44% after 20 days of drought stress. Si application proved beneficial in improving the performance of Kentucky bluegrass in the present study suggesting that manipulation of endogenous Si through genetic or biotechnological means may result in the development of drought resistance in grasses.

## 1. Introduction

Drought is one of the gravest threats to plants, and due to global warming; its prevalence is increasing worldwide [1]. Approximately one-third of the world land area is prone to drought, and, in China, this ratio is higher up to 47% [2]. Drought induces various changes in morphological, metabolic, or/and physiological functions of plant. At the initial phase of plant growth and establishment, it negatively affects both elongation and expansion growth [3, 4]. Reduced leaf growth and in turn the leaf areas and higher root/shoot ratio in response to drought has also been reported in many species [5]. Severe water stress poses injurious effects on plant water relations, photosynthesis, ion uptake, and nutrient metabolism and assimilates partitioning [5, 6]. Interrupted water supply from the xylem to the surrounding elongating cells under drought stress leads to loss of turgor and stomatal closure [7]. It also disturbs the photosynthetic apparatus through its interaction with UV or/and visible radiation [8]. Both stomatal and nonstomatal limitations are generally considered to be the main determinant of reduced photosynthesis under drought stress [6].

Silicon (Si) is the second most abundant element existing in the Earth's crust [9]. Although it is not considered as an essential element, nevertheless, there is increasing evidence regarding its beneficial effects on plant growth and development [10, 11]. Si acts as a physical or mechanical barrier in plants and not only acts as cell wall incustation, but is also actively involved in many metabolic and/or physiological processes. Many plants deprived of Si suffer significant reductions in growth and yield as well as increased susceptibility to both biotic and abiotic stresses [12]. Studies have shown that beneficial effects of Si are more prominent under stressful conditions as it can increase plant defense systems against salinity [13, 14] low [9] and high temperature [11], UV-radiation [15, 16] and heavy metal toxicity [17].

Si is also reported to increase drought tolerance in plants by maintaining leaf water potential, photosynthetic activity, stomatal conductance, leaves erectness, and structure of xylem vessels under high transpiration rates [10, 11, 18]. All these parameters have been widely used as physiological indicators for the selection of drought-tolerant plant materials [19]. Si can reduce the electrolyte leakage from rice leaves and therefore promote photosynthetic activity in plants grown under water deficit conditions [20]. Gong et al. [18] reported higher water use efficiency by application of Si in wheat. Gao et al. [21] reported that Si influences stomata movement and, therefore, affects transpiration rate through stomata. Matoh et al. [22] suggested that application of Si results in formation of a silica-cuticle double layer on leaf epidermal tissue, which is responsible for higher leaf water potential under water stress conditions. Endodermal tissues are known to accumulate large amounts of Si in drought tolerant cereal cultivars [23]. Results of Lux et al. [23] and Hattori et al. [24] revealed that Si plays a critical role in root growth and water movement from rhizosphere to roots under drought conditions in sorghum. Hattori et al. [11] ascribed the higher water flux to lowered hydraulic resistance resulting from higher root growth and water transport in silicon-applied sorghum under drought stress.

The amount of Si accumulation in the shoot through the roots varies between 0.1 and 10.0% [9], and such accumulations are reported to be higher in monocotyledons with respect to dicotyledons [25]. It has been reported that adding Si to monocots, especially Gramineae plants, ensured better growth and development due to large amounts of Si accumulation [26]. Being a member of Gramineae family, Kentucky bluegrass, which has a relatively excellent adaptability to various environments, is used often to cover barren soil and with an increasing interest in the quality of life and green environment; there has been an expansion of its utility and areas of use. Drought is known to limit the growth of turf grasses and can cause severe decline in its quality [19]. Despite the availability of volumetric information on role of Si regarding plant water relations and gas exchange in different field crops under stressful conditions [11, 13, 14, 23, 24], effect of Si has rarely been investigated on performance of Kentucky bluegrass under drought. It is hypothesized that Si application may improve growth and drought tolerance of Kentucky bluegrass by modulating associated morphophysiological changes and plant water relations. The present study intends to unravel the role of Si application in regulating drought tolerance, water relations, morphophysiological growth, and quality of Kentucky bluegrass. Optimization of Si application rate for Kentucky bluegrass under drought stress will be another objective of the study.

## 2. Materials and Methods

### 2.1. Plant Material and Treatments

Seeds of Kentucky bluegrass “Midnight” were sown in polyvinyl chloride (PVC) pots (16 cm diameter and 40 cm height) filled with 5.2 kg mixture of vermiculite and loam soil (1 : 4 v/v; with 625 mg kg^−1^ of effective Si) under glasshouse conditions. Midnight is well known, drought tolerant, and widely grown variety of Kentucky bluegrass. Seeds started germination after 10 days of sowing and plants were established for 2 months. Average daily day and night temperature was 25 ± 2°C and 15 ± 2°C, respectively. Relative humidity of 75 ± 5% and natural sunlight were maintained during the study. Plants were cut to 10 cm every week and were fertilized at fortnightly interval (17N-6P-10K) during the experimental period.

Drought stress and Si treatments were imposed after 2 months old plants cultured under glasshouse conditions. Drought stress was given by completely withholding irrigation for 20 days. Soil water contents were monitored on every 5 d intervals by TDR200 (Spectrum, USA) combined with weighting pots. Well-water controls were irrigated every day until water drained from the bottom of pot to maintain maximum soil water content. Plants were fertilized with four levels of Si (Na_2_SiO_3_ *·*9H_2_O) treatments after every 3 days with Hoagland's nutrient solution modified to supply Si at 0 mg L^−1^ (Si-0), 200 mg L^−1^ (Si-200), 400 mg L^−1^ (Si-400), and 800 mg L^−1^ (Si-800) under drought stress. Well-watered and drought stress treatments without Si application were also maintained for comparison. After 20 days of drought stress, the plants were rewatered to reach soil field capacity for the examination of recovery on the 7th and 14th days. Treatments were arranged in a completely randomized design with four replicates. Measurements were taken on every 5 d intervals, and then on the rewatered 7 d and 14 d. All studies were repeated 3 times during 2012 and 2013.

### 2.2. Data Collection

Net photosynthesis (*A*), transpiration rate (Tr), and stomatal conductance (*g*_*s*_) were determined using Li-6400 (Li-6400, LICOR, Inc., Lincoln, NB, USA) following the method described in Hu et al. [27]. Six individual leaves (second fully expand from the top) were taken from each pot and were placed in leaf chamber with a built-in red and blue light source of the Li-6400, and all measurements were taken on at the level of 800 *μ*mol m^−2^ s^−1^ photosynthetic photon flux density, which was the light saturation point for Kentucky bluegrass leaves. Leaf instantaneous water use efficiency (IWUE) was calculated by dividing *A* by Tr [28].

Soil water contents (SWC) were measured using time domain reflectometry (TDR200, Soil Moisture Equipment, Spectrum, USA) by inserting the 20-cm-long wave guide probe to monitor the soil water deficit in the top 20-cm soil profile. Leaf relative water content (RWC) of fully expanded leaves was determined based on fresh (FW), turgid (TW), and dry weights (DW) using the following formula: RWC (%) = [(FW − DW)/(TW − DW)] × 100. Leaf fresh weight was immediately weighed (Mettler AE260 balance, USA) after being excised from the plants and then was soaked in deionized water for 6 h at room temperature 25 ± 1°C. Leaf samples were then blotted dry and immediately weighed for determination of TW. Samples were then dried in an oven at 80°C for 72 h and weighed again for DW. The relative growth rate was calculated according to average daily growth rate compared to the control. Leaf blade width and length were measured visually by a ruler with the minimum scale of 0.01 cm. Shoot and root biomass was determined from washed samples oven-dried at 60~65°C for 48 h and then weighed and root/shoot ratio was computed. Quality of Kentucky bluegrass was represented by turf green color measured by SPAD502 (Minolta, Japan) combined with visual rating from 1–9 with 6 being considered the minimal acceptable level and 9 being healthy plants with dark-green and turgid leaf blades and a dense turf canopy [29]. The samples of leaves were ground and passed through a 20 mesh screen after being dried at 80°C for 36 h. The total contents of nitrogen (N) and organic carbon (C) were determined by the semimicro-Kjeldahl method and the rapid dichromate oxidation technique [30], respectively. The total C to N ratio (C : N) (g g^−1^ DW) was calculated as an estimate for the long term nitrogen use efficiency [31].

### 2.3. Statistical Analysis

Studies were carried out following completely randomized design with four replications and repeated three times. Since the results of all runs of whole experiment were statistically similar (*P* ⩽ 0.05), data were pooled for further statistical analyses. Data collected were subjected to statistical analysis by analysis of variance using the computer software SPSS (*version 12*, SPSS, Chicago, IL, USA). The mean values were compared with the least significance difference test at 0.05 probability level. The relationships between different attributes were evaluated by using hyperbola regression analysis.

## 3. Results

**Figure 1:**
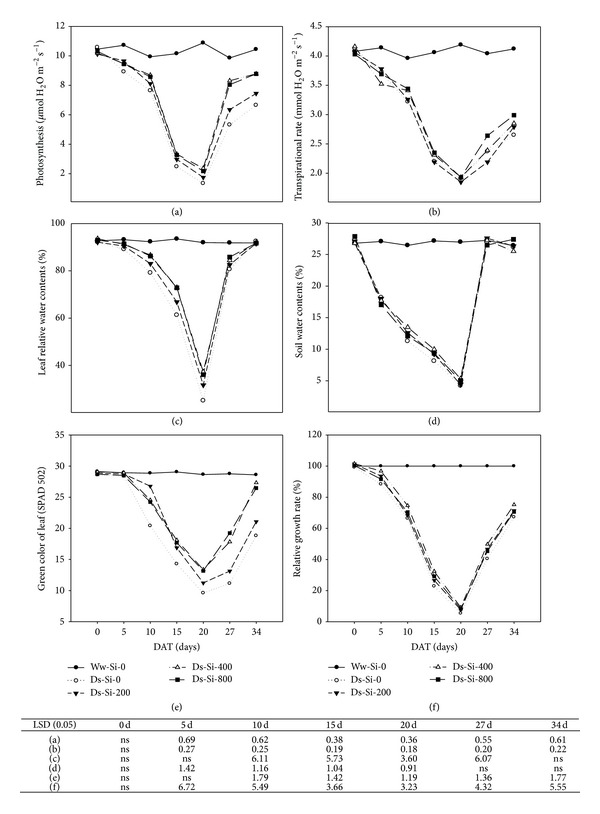
Influence of silicon application on (a) photosynthesis, (b) transpiration rate, (c) leaf relative water content, (d) soil water content, (e) green color of leaf, and (f) relative growth rate of Kentucky bluegrass under drought stress. ns: nonsignificant; DAT: days after treatment; Ww: well watered; Ds: drought stress; Si-0, Si-200, Si-400, and Si-800 means Si application at 0, 200, 400, and 800 mg L^−1^, respectively. 0–20 DAT: drought stress period; 20–34 DAT: recovery stage.

Drought stress significantly decreased the net photosynthesis (*A*) and transpiration rate (Tr) of Kentucky bluegrass as compared to well watered treatment (Figures 1(a) and 1(b)). When plants were subjected to drought, both attributes were progressively decreased with the passage of time until recovery stage. After 20 d of drought stress, 88% and 55% reductions in *A* and Tr, respectively, were recorded in treatments, where no Si was applied. Application of Si at 400 and 800 mg L^−1^ showed 44% and 39% increase in *A* under drought stress; nevertheless, these both treatments could not significantly alter the Tr as compared to no Si application under drought. When plants were rewatered after drought stress, *A* and Tr were increased and such an increase was higher in Si applied treatments.

Leaf water content (RWC; Figure 1(c)), soil water contents (SWC; Figure 1(d)), leaf green color (LGC; Figure 1(e)), and relative growth rate (RGR; Figure 1(f)) were almost constant during experimental period under well watered conditions. However, these variables were severely declined with progressive drought and the decline was more pronounced when no Si was applied. Fertilization of Si remained beneficial under drought and Si applied at 400 mg L^−1^ recorded an increase of 33, 21, 42, and 22% in RWC, SWC, LGC, and RGR, respectively, as compared to no Si application at 20 d after drought stress.

**Figure 2:**
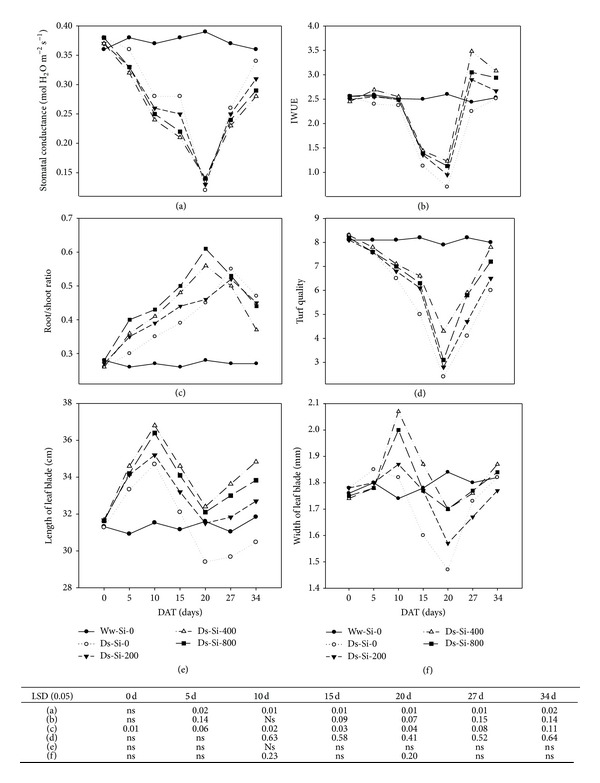
Influence of silicon application on (a) stomatal conductance, (b) IWUE, (c) root/shoot ratio, (d) turf quality, (e) leaf blade length, and (f) leaf blade width of Kentucky bluegrass under drought stress. ns: nonsignificant; DAT: days after treatment; Ww: well watered; Ds: drought stress; Si-0, Si-200, Si-400, and Si-800 means Si application at 0, 200, 400, and 800 mg L^−1^, respectively. From *t* = 0–20 days: drought stress period, after 20 days: recovery stage.

The initial level of stomatal conductance (*g*_*s*_; Figure 2(a)) and instantaneous water use efficiency (IWUE; Figure 2(b)) was almost similar for all treatments. Nonetheless, when Kentucky bluegrass plants were subjected to drought, *g*_*s*_ and IWUE were decreased with the passage of time, until plants were rewatered. Application of Si resulted in less *g*_*s*_ under stress conditions. IWUE of Kentucky bluegrass was significantly increased in response to Si application during whole experiment. When these Kentucky bluegrass plants were rewatered after stress, they showed higher IWUE even than well watered plants.

The root/shoot ratio (Figure 2(c)) of Kentucky bluegrass was increased under drought conditions to a significant level. Under no Si application, the root/shoot ratio of Kentucky bluegrass was 13, 23, 34, and 38% higher after 5, 10, 15, and 20 d of drought stress, respectively, as compared to well watered treatment. Increase in root/shoot ratio of Kentucky bluegrass was proportional to rate of Si application and Si applied at 800 mg L^−1^ recorded highest root/shoot ratio. Drought stress also hampered the turf quality (Figure 2(d)), and Si application appeared beneficial in lowering such ill effects. The initial level of turf quality was approximately 8.0 for all treatments, which declined to below acceptable level after 20 d of stress. Si application at 400 mg L^−1^ remained superior to the rest of treatment, as this treatment improved the turf quality by 44% as compared to no Si application under drought stress. Leaf blade (Figures 2(e) and 2(f)) of Kentucky bluegrass responded differentially to drought stress as well as Si application, but these differences were not to a significant level. Length as well as width of leaf blade was increased under drought up till 10 d after stress, which declined afterward. Application of Si proved beneficial as it increased size of leaf blade in terms of length and width.

Significant increase in carbon : nitrogen (C : N) ratio of Kentucky bluegrass leaves was observed, when plants were subjected to drought stress (Table 1). Such an increase was more pronounced with the passage of time until plants were rewatered. After 15 d of drought stress, C : N ratio of Kentucky bluegrass leaves was increased by 40%; nevertheless, Si application at 400 mg L^−1^ was more effective in decreasing (15%) the C : N ratio of Kentucky bluegrass under stressful conditions.

**Figure 3:**
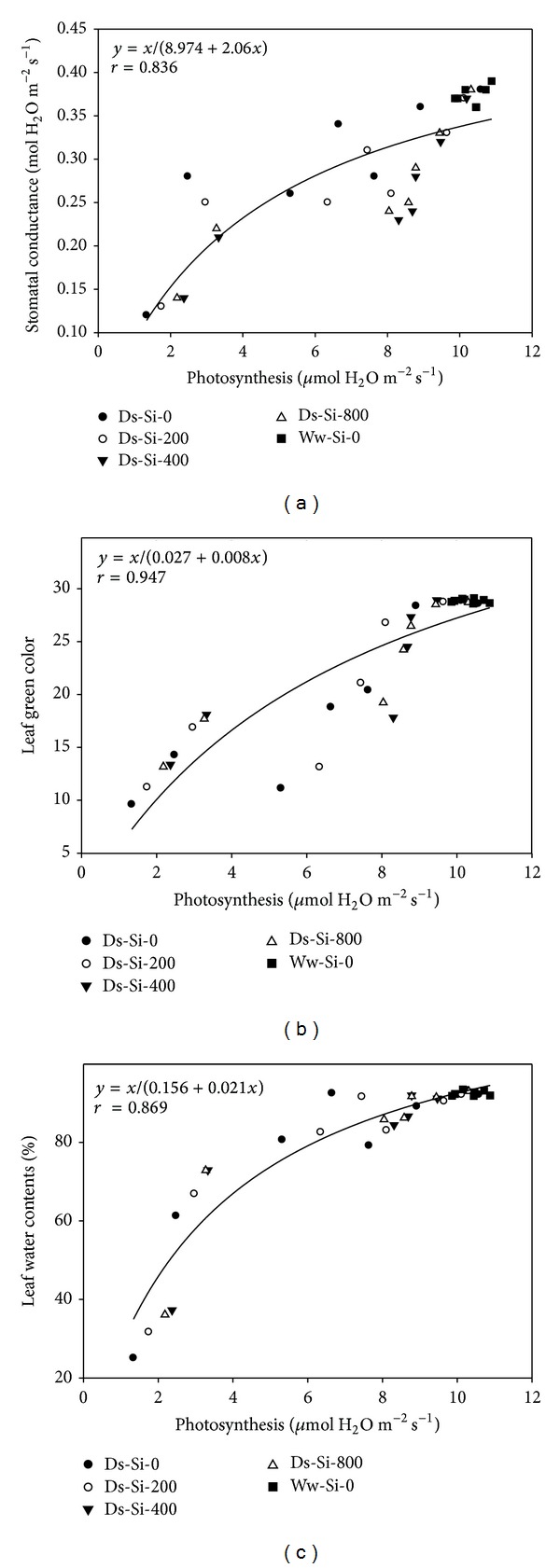
Relationship of net photosynthesis with (a) stomatal conductance, (b) leaf green color, and (c) leaf water contents using hyperbola regression analysis.

**Figure 4:**
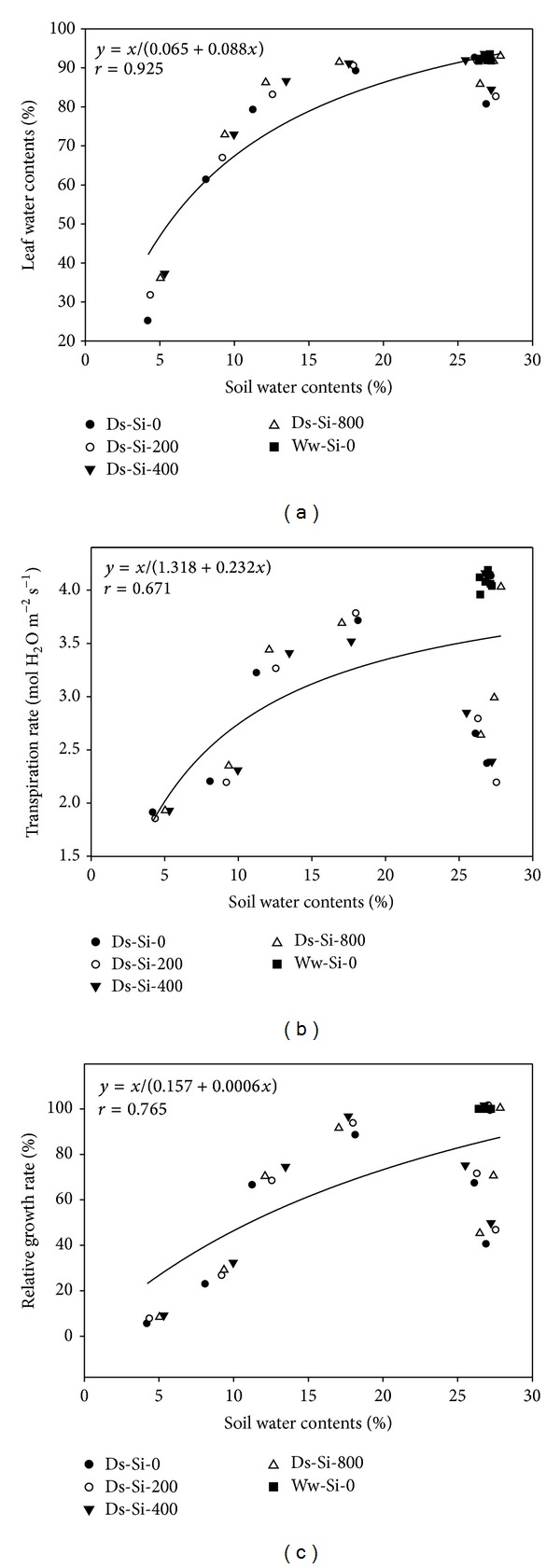
Relationship of soil water content with (a) leaf water content, (b) relative growth rate, and (c) transpiration rate using hyperbola regression analysis.

The relationship drawn between *A* and RWC, *A* and *g*_*s*_, and *A* and LGC showed the strong positive association of these attributes. Hyperbola regression analysis (nonlinear relationship) revealed 84%, 95%, and 87% variations for *A* and *g*_*s*_, *A* and LGC, and *A* and RWC, respectively (Figure 3). Likewise, positive relationship was found between SWC and Tr, SWC and RGR, and WC and RWC depicting 67, 77, and 93% variations, respectively (Figure 4).

## 4. Discussion

Drought stress hampered all the morphophysiological attributes, water relations, and turf quality of Kentucky bluegrass; nonetheless, Si application was beneficial in alleviating the adverse effects of drought stress. Previously many studies have documented plant growth improvement by Si application under drought conditions in many species including wheat [10, 18], rice [32], sorghum [23, 24], and soybean [15] plants. Eneji et al. [33] also reported improvements in growth and nutrient use of four grass species (rhodes grass, timothy grass, sudangrass and tall fescue) following Si application under water deficit conditions.

Reduced photosynthesis is the major effect caused by drought, which presumably arises by a decreased leaf expansion and impaired photosynthetic machinery [34]. Farooq et al. [6] stated that stomatal and nonstomatal limitations are the main determinant of reduced photosynthesis under water limited condition. In present study, there was strong positive relationship of photosynthesis with stomatal conductance as well as leaf green color (Figure 3), as both attributes were severely affected by drought stress; therefore, reduction in stomatal conductance and leaf green color might be ascribed as the major cause of reduced photosynthesis. Reddy et al. [35] reported that the decrease in photosynthesis under drought through metabolic impairment is more complex than stomatal conductance and mainly it is through reduced photosynthetic pigment contents in sunflower.

In present study, drought severely diminished plant water relation by decreasing leaf water potential, stomatal conduction, and transpiration rate. Such reductions could be attributed to decrease in soil water potential (Figure 1) which made water unavailable to root systems for compensating water loss by transpiration. Soil water contents were strongly linked with leaf water potential and transpiration rate, which justified our assumption (Figure 4). While working on* Hibiscus rosa-sinensis*, Egilla et al. [36] observed that relative water content, stomatal conductance, turgor potential, transpiration, and water use efficiency were severely decreased under drought stress. Siddique et al. [37] proposed that concomitant increase in leaf temperature upon exposure to drought stress substantially decreased the relative water content, leaf water potential, and transpiration rate in wheat plants.

Si application significantly increased the photosynthetic rate, leaf water potential, relative growth rate, leaf green color, and water use efficiency of Kentucky bluegrass under drought stress. This might be attributed to improved water uptake, root growth, and leaf erectness by application of Si [11, 18] that maintained higher leaf water potential, leaf green color, and higher photosynthesis in the present study. Previously, Gong et al. [18] reported that addition of 7.14 mmol Na_2_SiO_3_ per 8 kg of soil increased (2.7%) the leaf relative water content and leaf water potential (0.4 MPa) in wheat under drought conditions. Gong et al. [10] found that application of 2.11 mmol Na_2_SiO_3_ increased net assimilation rate by ~37 mmol C m^−2^ s^−1^ under water deficit conditions in wheat. Hattori et al. [11] observed that growth rate of Si-applied (1.66 mM K_2_SiO_3_) sorghum was higher under drought conditions as compared to control. Accumulation of silicon in the leaves increases leaf blade erectness, which in turns facilitates light penetration, decreases transpiration, and promotes photosynthesis. Higher water use efficiency in Si applied Kentucky bluegrass might be ascribed to higher photosynthesis and growth rate and less transpiration rate. Gao et al. [21] also recorded that Si improved the water use efficiency in maize plant.

Reduced turf quality of Kentucky bluegrass under drought stress might be due to withering of leaves caused by decease in relative water contents. Liu et al. [19] have also reported that water shortage in soil can reduce the quality of Kentucky bluegrass leaves. In present study, increase in root/shoot ratio and decrease in leaf blade size were observed under drought stress. Tahir et al. [38] and Jaleel et al. [39] have also observed increased root growth due to water stress in sunflower and* Catharanthus roseus *plants, respectively. Previously some authors have related such increase in root/shoot ratio to ABA content of roots and shoots under drought conditions [40, 41]. Increase in C : N ratio of Kentucky bluegrass leaves by the exposure of drought stress is also evident form the results, which might be attributes to decrease in leaf N contents. A reduced transpiration rate due to water deficit reduces the nutrient absorption and efficiency of their utilization. Less water availability under drought conditions generally limits total nutrient uptake and diminishes tissue concentrations in plants. Furthermore, such effects may also be related to limited availability of energy for assimilation of NO_3_^−^/NH_4_^+^ under drought conditions [6].

## 5. Conclusion

Drought stress posed strong negative effects on growth and quality of Kentucky bluegrass. Nevertheless, Si application remained effective in alleviating the negative effects of drought stress. Taking in conjunction the results of the present study, the enhanced performance of Si applied Kentucky bluegrass seems to arise from (1) improved water relations, (2) better gas exchange, and (3) increased morhophysiological functions. Our study provides an insight and is a step forward in establishing the role of Si for improving performance of drought-stressed Kentucky bluegrass. At the same time, it implies that Si application rate of 400 mg L^−1^ is superior in terms of all studied attributes under drought stress as well as after recovery stage. Our results justified the beneficial role of Si for Kentucky bluegrass under drought stress and suggested that manipulation of endogenous Si through genetic or biotechnological means may result in the development of drought resistance in Kentucky bluegrass.

## Figures and Tables

**Table 1:** Influence of silicon application on C : N ratio of Kentucky bluegrass leaves under drought stress.

Treatment	Days after treatment			
	0 d	10 d	15 d	27 d
Ww-Si-0	17.36^a^	17.86^b^	17.14^c^	17.94^d^
Ds-Si-0	17.88^a^	21.98^a^	28.73^a^	24.83^a^
Ds-Si-200	17.93^a^	21.25^a^	27.52^ab^	22.65^ab^
Ds-Si-400	17.40^a^	19.29^ab^	24.56^b^	19.64^cd^
Ds-Si-800	17.65^a^	20.17^ab^	26.37^ab^	20.95^bc^

LSD (0.05)	ns	2.82	3.53	2.99

Means sharing different latters are significant at LSD (0.05). ns: nonsignificant; Ww: well watered; Ds: drought stress; Si-0, Si-200, Si-400, and Si-800 means Si application at 0, 200, 400, and 800 mg L^−1^, respectively. From *t* = 0–20 d: drought stress period, after 20 days: recovery stage.

## Abbreviations

*A*: Photosynthetic rate
C : N: Carbon/nitrogen
*g*_*s*_: Stomatal conductance
IWUE: Instantaneous water use efficiency
LGC: Leaf green color
LSD: Least significant difference
LWC: Leaf water content
Si: Silicon
SWC: Soil water content
Tr: Transpiration rate.

## References

[1] Yang S, Vanderbeld B, Wan J, Huang Y (2010). Narrowing down the targets: towards successful genetic engineering of drought-tolerant crops. *Molecular Plant*.

[2] Wei L, Jia L, Hu X, Zhao F (1997). Advances in studies on the physiology and biochemistry of maize drought resistance. *Agricultural Research in the Arid Areas*.

[3] Kusaka M, Ohta M, Fujimura T (2005). Contribution of inorganic components to osmotic adjustment and leaf folding for drought tolerance in pearl millet. *Physiologia Plantarum*.

[4] Shao HB, Chu LY, Shao MA, Jaleel CA, Hong-mei M (2008). Higher plant antioxidants and redox signaling under environmental stresses. *Comptes Rendus—Biologies*.

[5] Jaleel CA, Manivannan P, Wahid A (2009). Drought stress in plants: a review on morphological characteristics and pigments composition. *International Journal of Agriculture and Biology*.

[6] Farooq M, Wahid A, Kobayashi N, Fujita D, Basra SMA (2009). Plant drought stress: effects, mechanisms and management. *Agronomy for Sustainable Development*.

[7] Taiz L, Zeiger E (2006). *Plant Physiology*.

[8] García-Plazaola JI, Becerril JM (2000). Effects of drought on photoprotective mechanisms in European beech (*Fagus sylvatica* L.) seedlings from different provenances. *Trees*.

[9] Epstein E (1999). Silicon. *Annual Review of Plant Biology and Plant Molecular Biology*.

[10] Gong H, Zhu X, Chen K, Wang S, Zhang C (2005). Silicon alleviates oxidative damage of wheat plants in pots under drought. *Plant Science*.

[11] Hattori T, Inanaga S, Araki H (2005). Application of silicon enhanced drought tolerance in *Sorghum bicolor*. *Physiologia Plantarum*.

[12] Datnoff LE, Snyder GH, Korndörfer GH (2001). *Silicon in Agriculture*.

[13] Liang Y, Chen Q, Liu Q, Zhang W, Ding R (2003). Exogenous silicon (Si) increases antioxidant enzyme activity and reduces lipid peroxidation in roots of salt-stressed barley (*Hordeum vulgare* L.). *Journal of Plant Physiology*.

[14] Biel KY, Matichenkov VV, Fomina IR, Martirosyan DM (2008). Protective role of silicon in living systems. *Functional Foods for Chronic*.

[15] Shen X, Zhou Y, Duan L, Li Z, Eneji AE, Li J (2010). Silicon effects on photosynthesis and antioxidant parameters of soybean seedlings under drought and ultraviolet-B radiation. *Journal of Plant Physiology*.

[16] Fang CX, Wang QS, Yu Y, Huang LK, Wu X, Lin WX (2011). Silicon and its uptaking gene Lsi1 in regulation of rice UV-B tolerance. *Acta Agronomica Sinica*.

[17] Shi X, Zhang C, Wang H, Zhang F (2005). Effect of Si on the distribution of Cd in rice seedlings. *Plant and Soil*.

[18] Gong H, Chen K, Chen G, Wang S, Zhang C (2003). Effects of silicon on growth of wheat under drought. *Journal of Plant Nutrition*.

[19] Liu J, Xie X, Du J, Sun J, Bai X (2008). Effects of simultaneous drought and heat stress on *Kentucky bluegrass*. *Scientia Horticulturae*.

[20] Agarie S, Uchida H, Agata W, Kubota F, Kaufman PB (1998). Effects of silicon on transpiration and leaf conductance in rice plants (*Oryza saliva* L.). *Plant Production Science*.

[21] Gao X, Zou C, Wang L, Zhang F (2006). Silicon decreases transpiration rate and conductance from stomata of maize plants. *Journal of Plant Nutrition*.

[22] Matoh T, Murata S, Takahashi E (1991). Effect of silicate application on photosynthesis of rice plants. *Japan Journal of Soil Science and Plant Nutrition*.

[23] Lux A, Luxová M, Abe J, Morita S, Inanaga S (2003). Silicification of bamboo (*Phyllostachys heterocycla* Mitf.) root and leaf. *Plant and Soil*.

[24] Hattori T, Inanaga S, Tanimoto E, Lux A, Luxová M, Sugimoto Y (2003). Silicon-induced changes in viscoelastic properties of Sorghum root cell walls. *Plant and Cell Physiology*.

[25] Ma JF, Yamaji N (2006). Silicon uptake and accumulation in higher plants. *Trends in Plant Science*.

[26] Mitani N, Jian FM (2005). Uptake system of silicon in different plant species. *Journal of Experimental Botany*.

[27] Hu L, Wang Z, Huang B (2010). Diffusion limitations and metabolic factors associated with inhibition and recovery of photosynthesis from drought stress in a C_3_ perennial grass species. *Physiologia Plantarum*.

[28] Robredo A, Pérez-López U, de la Maza HS (2007). Elevated CO_2_ alleviates the impact of drought on barley improving water status by lowering stomatal conductance and delaying its effects on photosynthesis. *Environmental and Experimental Botany*.

[29] Turgeon AJ (2008). *Turfgrass Management*.

[30] Nelson DW, Sommers LE, Page AL (1982). Total carbon, organic carbon and organic matter. *Methods of Soil Analysis. vol 2. Chemical and Microbiological Properties*.

[31] Livingston NJ, Guy RD, Sun ZJ, Ethier GJ (1999). The effects of nitrogen stress on the stable carbon isotope composition, productivity and water use efficiency of white spruce (*Picea glauca* (Moench) Voss) seedlings. *Plant, Cell and Environment*.

[32] Chen W, Yao X, Cai K, Chen J (2011). Silicon alleviates drought stress of rice plants by improving plant water status, photosynthesis and mineral nutrient absorption. *Biological Trace Element Research*.

[33] Eneji AE, Inanaga S, Muranaka S (2008). Growth and nutrient use in four grasses under drought stress as mediated by silicon fertilizers. *Journal of Plant Nutrition*.

[34] Wahid A, Rasul E, Pessarakli M (2005). Photosynthesis in leaf, stem, flower and fruit. *Handbook of Photosynthesis*.

[35] Reddy AR, Chaitanya KV, Vivekanandan M (2004). Drought-induced responses of photosynthesis and antioxidant metabolism in higher plants. *Journal of Plant Physiology*.

[36] Egilla JN, Davies FT, Boutton TW (2005). Drought stress influences leaf water content, photosynthesis, and water-use efficiency of *Hibiscus rosa-sinensis* at three potassium concentrations. *Photosynthetica*.

[37] Siddique MRB, Hamid A, Islam MS (2001). Drought stress effects on water relations of wheat. *Botanical Bulletin of Academia Sinica*.

[38] Tahir MHN, Imran M, Hussain MK (2002). Evaluation of sunflower (*Helianthus annuus* L.) inbred lines for drought tolerance. *International Journal of Agriculture and Biology*.

[39] Jaleel CA, Manivannan P, Lakshmanan GMA, Gomathinayagam M, Panneerselvam R (2008). Alterations in morphological parameters and photosynthetic pigment responses of *Catharanthus roseus* under soil water deficits. *Colloids and Surfaces B: Biointerfaces*.

[40] Sharp RE, Lenoble ME (2002). ABA, ethylene and the control of shoot and root growth under water stress. *Journal of Experimental Botany*.

[41] Manivannan P, Jaleel CA, Sankar B (2007). Growth, biochemical modifications and proline metabolism in *Helianthus annuus* L. as induced by drought stress. *Colloids and Surfaces B: Biointerfaces*.

